# A Modified QuEChERS Method for Determination of Pyrethroid Residues in Traditional Chinese Medicine Oral Liquids by High-Performance Liquid Chromatography

**DOI:** 10.3390/molecules24081470

**Published:** 2019-04-13

**Authors:** Yuge Hou, Hong Chen, Xin Li, Yiyi Liao, Makoto Tsunoda, Yingxia Zhang, Shiming Deng, Yanting Song

**Affiliations:** 1 Key Laboratory of Tropical Biological Resources of Ministry of Education, Department of Pharmaceutical Sciences, School of Life and Pharmaceutical Sciences, Hainan University, Haikou 570228, China; hygnlnl@163.com (Y.H.); cheaterking666@163.com (H.C.); iamlixin96@163.com (X.L.); liaoyylyy@163.com (Y.L.); yingxiazhang@hotmail.com (Y.Z.); dsm701@126.com (S.D.); 2 Graduate School of Pharmaceutical Sciences, The University of Tokyo, 7-3-1 Hongo, Bunkyo-ku, Tokyo 113-0033, Japan; makotot@mol.f.u-tokyo.ac.jp

**Keywords:** traditional Chinese medicine oral liquid, pyrethroid pesticide, QuEChERS, high-performance liquid chromatography

## Abstract

Pyrethroid residues in traditional Chinese medicines have been a serious threat to the health and treatment of patients. However, because of the matrix complexity of traditional Chinese medicine, the detection of pyrethroid residues remains a challenge. Therefore, we developed a QuEChERS method coupled with high-performance liquid chromatography and ultraviolet detection (HPLC-UV) for the determination of pyrethroid pesticides in three kinds of traditional Chinese medicine oral liquid preparations, and we investigated and optimized the extraction conditions. The matrix effect was estimated in the organic solvent and the actual samples by comparing the slopes of calibration curves, and the results showed that the matrix effect is not significant when using the modified QuEChERS method. The pyrethroid pesticides could be completely separated in 30 min. The linear correlation coefficients were more than 0.999, and the recoveries of all the pyrethroid pesticides ranged from 87.2% to 104.8%. The intra-day precisions (n = 5) were 2.44–4.62%, and the inter-day precisions (n = 5) were 1.06–3.02%. Moreover, the limits of detection were in the range of 0.007–0.018 ng mL^−1^, while the limits of quantitation were in the range of 0.022–0.057 ng mL^−1^. This simple, low-cost, and highly sensitive analytical method can be a potential tool for the analysis of pyrethroid residues in traditional Chinese medicine oral liquid preparations.

## 1. Introduction

Pyrethroids have been considered one of the sources of agricultural pollution because of their widespread use, and even abuse [1]. Additionally, they may be a potential threat to human health, as prolonged skin contact and misuse can cause symptoms of poisoning [2]. The mechanism of poisoning is mainly to influence the conduction of axons and cause muscle spasm [3,4]. Traditional Chinese medicine (TCM) refers to substances that are used to prevent, treat, and diagnose diseases and these substances have rehabilitation and health care functions under the guidance of TCM theory. Traditional Chinese medicines are mainly derived from natural medicines and processed products [5]. During the cultivation of Chinese herbal medicines, the use of pesticides may cause accumulation, which results in toxicity to the environment and humans. Because each traditional Chinese medicine preparation has two or more principal herbal ingredients, the complex matrix will interfere with the determination of trace pyrethroid residues. Therefore, to establish an analytical method for the detection of pyrethroids in TCM oral liquids will be very challenging.

Until now, many researchers have focused on the detection of pyrethroid pesticides [6,7,8]. In these studies, various sample pretreatment methods including dispersive liquid-liquid microextraction (DLLME), solid phase extraction (SPE), molecular imprinting (MIP), and magnetic solid phase extraction (MSPE) have been employed. Yu et al. compared the pyrethroid residues (bifenthrin, cypermethrin, decamethrin, fenvalerate, fenpropathrin, and Permethrin) in fruit peels and flesh. He synthesized polystyrene-coated nanoparticles, and performed an MSPE procedure for determination [7]. Suthasinee Boonchiangma et al. determined six pyrethroid residues in various fruit juices by applying dispersive liquid-liquid microextraction (DLLME) coupled to high-performance liquid chromatography (HPLC) with ultraviolet (UV) detection [8]. However, these methods are laborious and time-consuming, and therefore, could not achieve the requirements for rapid and sensitive analysis of pyrethroid pesticides.

The QuEChERS method involves microextraction with an organic solvent and purification with a dispersed solid phase microextraction (SPME) [9], and has been applied for the determination of trace compounds in complex matrixes such as fruits [10], vegetables [11], and biological samples [12]. Abdalrahman achieved high recoveries by using 1% acetic acid/acetonitrile as an extraction solvent when using the improved QuEChERS method combined with HPLC-DAD to determine the dissipation kinetics and residual levels of thiamethoxam in potato and soil under field ecosystem [13]. Melo et al. evaluated the trace levels of 13 pesticides in tomatoes, using an optimized QuEChERS method coupled with DLLME by HPLC [14]. However, the QuEChERS method has rarely been utilized for the determination of pesticide residue in TCM oral liquid samples. Due to the large-scale use of typical pyrethroids such as transfluthrin, fenpropathrin, fenvalerate, etofenprox, and silafluofen in daily life, the QuEChERS extraction and chromatographic conditions were investigated, and a modified QuEChERS method coupled with high-performance liquid chromatography (HPLC) was developed for the analysis of these pyrethroid pesticide residues in TCM oral liquid samples. In this study, three pesticide-free TCM oral liquids named Qutanling (composed of Succus bambusaes and *Houttuynia cordata* Thunb, used for cough and asthma), Yimucao (composed of *Leonurus japonicus* Houtt., used for irregular menstruation and postpartum lochia), and Shengmaiyin (composed of *Codonopsis pilosula* (Franch.) Nannf., *Ophiopogon japonicus* (L. f) Ker-Gawl., and *Schisandra chinensis* (Turcz.) Baill., used for myocardial infarction and arrhythmia) [5] were investigated.

## 2. Results and Discussion

### 2.1. Optimization of the QuEChERS Method

The optimization of this method was conducted by way of a single-factor experiment. 5 mL TCM oral liquid spiked with 100 ng mL^−1^ pyrethroids was extracted and purified and finally diluted with 100 μL of acetonitrile.

#### 2.1.1. Extraction Solvent Selection

**Figure 1:**
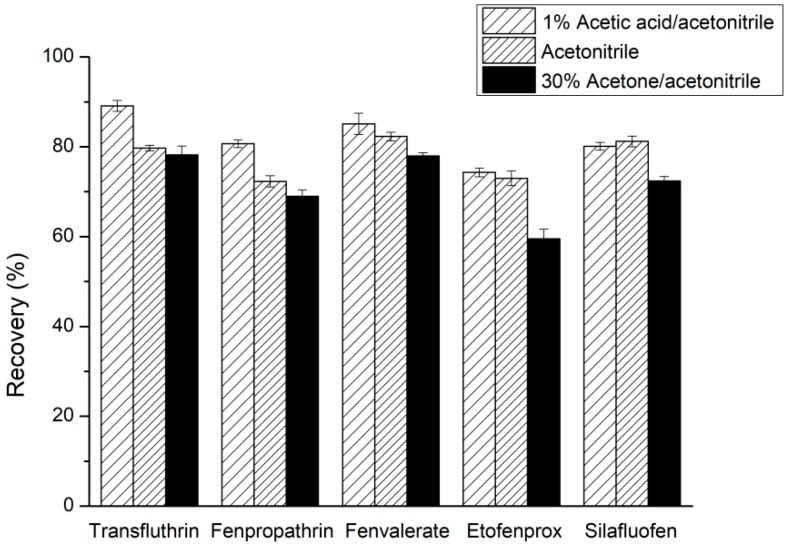
Effect of extraction solvent type on the recoveries of five pyrethroids.

Three types of extraction solvent (including acetonitrile–acetone (70:30, *v*/*v*) solution, acetonitrile containing 1% acetic acid, and acetonitrile) were investigated for the extraction of the five pyrethroid pesticide residues from TCM oral liquids. As shown in Figure 1, when acetonitrile-acetone was used as an extraction solvent, the extract had a deep yellow color, contained many impurities, and was difficult to purify. When the extraction solvent was acetonitrile containing 1% acetic acid, highest recoveries were obtained. The pyrethroids are faintly acid, and 1% acetic acid (*v*/*v*) will increase the solubility of pyrethroids in acetonitrile solutions; this resulted in higher recoveries [15].

#### 2.1.2. Optimization of the Extraction Solvent Amount

**Figure 2:**
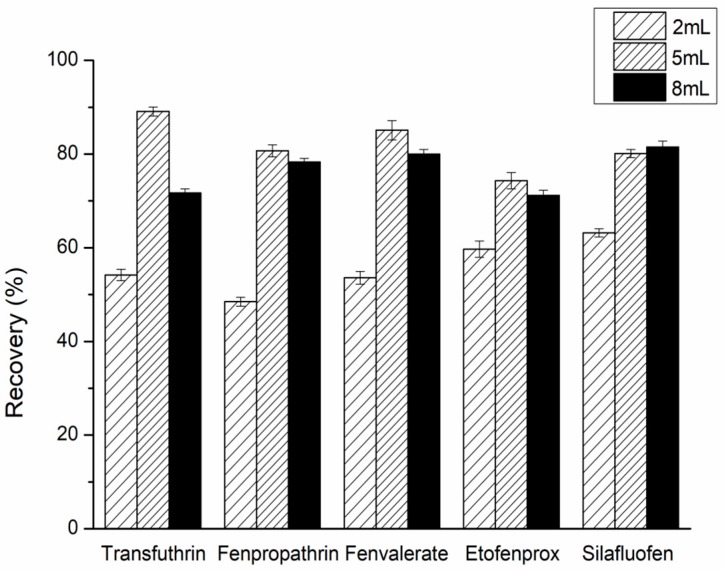
Effect of extraction solvent amount on the recoveries of five pyrethroids.

The amount of acetonitrile containing 1% acetic acid as an extraction solvent greatly influences the extraction efficiency. In this research, the effect of the amount of acetonitrile (2, 5, and 8 mL) was investigated. As depicted in Figure 2, the highest recovery rate was achieved when 5 mL of extraction solvent was used [16].

#### 2.1.3. Selection of the Purification Agent

**Figure 3:**
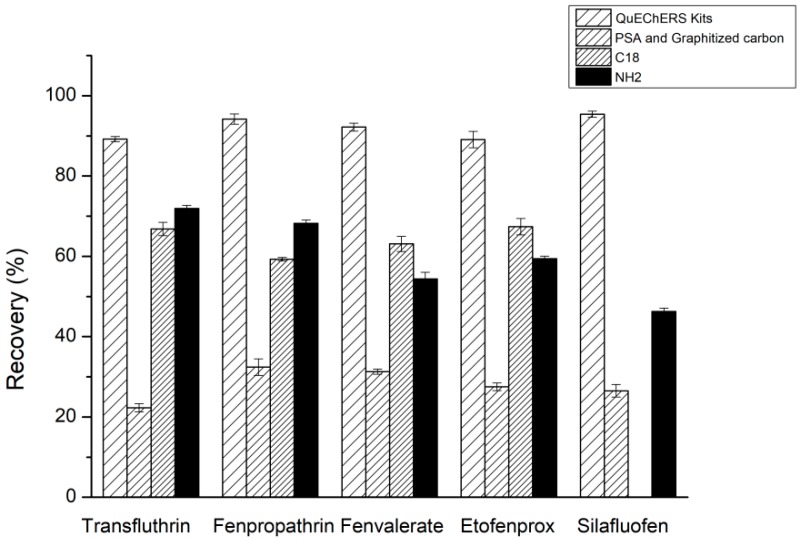
Effect of purification agent type on the recoveries of five pyrethroids.

The matrix of a TCM oral liquid sample is complex, and sugars and other impurities remain after extraction. The use of suitable cleaning agents can allow us to effectively detect the pesticide residues in the sample. The effects of PSA and graphitized carbon black, ProElut NH2, ProElut C18, and QuEChERS Extraction Kits (containing MgSO_4_, PSA, and C_18_) as dispersive solid phase extraction materials were investigated. As presented in Figure 3, the use of QuEChERS Kits could achieve high recoveries of the five pyrethroids and effectively eliminate the interference of lipids and sterols. PSA could not retain the pyrethroid pesticides while it could effectively remove polar interfering substances [17]. Therefore, the QuEChERS Kits were selected as the dispersive solid-phase extraction material to purify the acetonitrile extract.

#### 2.1.4. Selection of Added Salt

**Figure 4:**
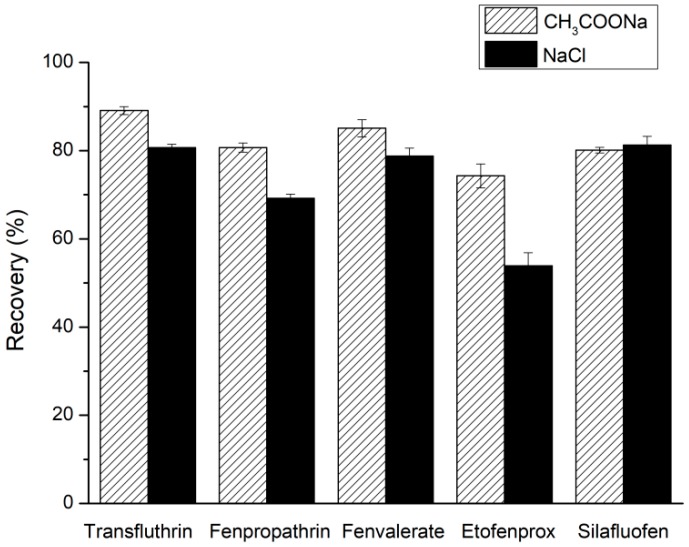
Effect of added salt type on the recoveries of five pyrethroids.

Anhydrous MgSO_4_ can be used to reduce the aqueous phase by hydration, and salt was added to promote the salting-out effect. In this study, the extraction efficiencies of two salts, CH_3_COONa and NaCl, were compared. When CH_3_COONa was used, higher recoveries were achieved for most of the pyrethroids (Figure 4). The addition of CH_3_COONa into acetonitrile extractant containing 1% acetic acid could result in a pH difference between the acetonitrile extraction phase and the aqueous phase, which would enhance the salting-out effect [15]. Therefore, CH_3_COONa was added during the extraction.

### 2.2. Method Validation

The limits of detection (LOD) and limits of quantification (LOQ) were determined on the basis of a signal to noise ratio of 3 (S/N = 3) and signal to noise ratio of 10 (S/N = 10), respectively. As shown in Table 1, the LOD of the five pyrethroid pesticides were between 0.007 and 0.018 ng mL^−1^, while the LOQ values were in the range of 0.022–0.057 ng mL^−1^. The linearity was within the range of 2–200 ng mL^−1^. The correlation coefficients for the five pyrethroids were higher than 0.999. The intra-day and inter-day precisions were obtained by analyzing three TCM oral liquid samples with spiked concentration of 20 ng mL^−1^ five times in a day and on five different days, respectively. The intra-day precisions (*n* = 5) were 2.44–4.62%, and the inter-day precisions (*n* = 5) were 1.06–3.02%. Accuracy was evaluated by analyzing the TCM oral liquid blank samples spiked with three different concentrations of the five pyrethroids (20, 50, and 100 ng mL^−1^). The recoveries for the five pyrethroids ranged from 87.2% to 104.8%. The enrichment factor of this method is between 45.16 and 51.07 (see Table 2).

**Figure 5:**
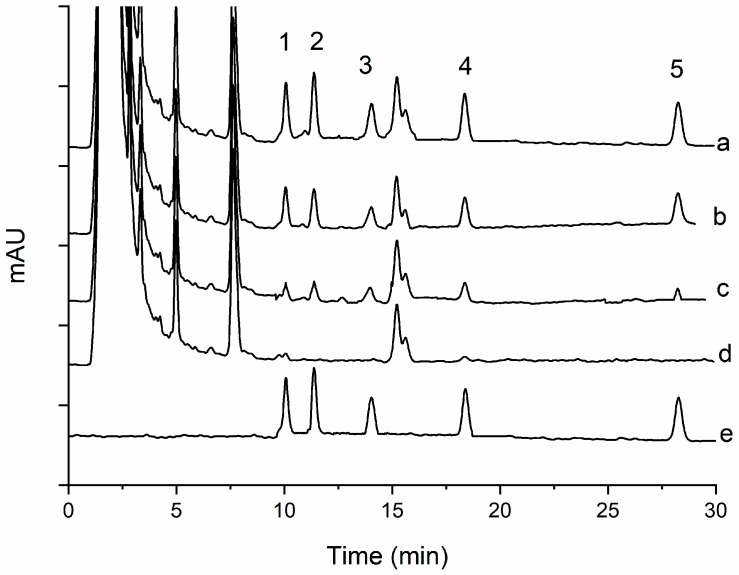
Typical chromatograms of five pyrethroids in oral qutanling liquids under optimum conditions: (1) transfluthrin, (2) fenpropathrin, (3) fenvalerate, (4) etofenprox, and (5) silafluofen. In chromatograms (a, c, d), the spiked levels are 100, 20, and 0 ng mL^−1^, and (b, e) show the standard solution in blank matrix and acetonitrile.

**Figure 6:**
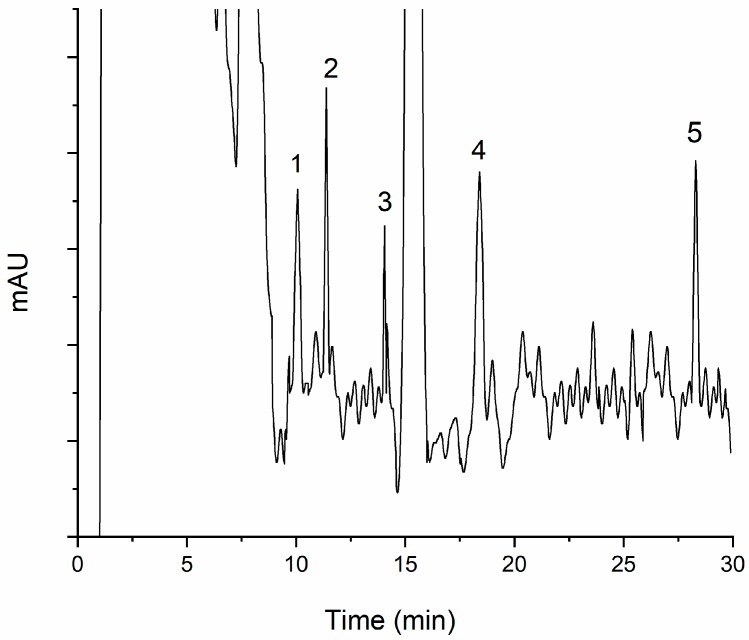
Typical chromatogram of five pyrethroids in Qutanling oral liquids under optimum conditions: (1) transfluthrin, (2) fenpropathrin, (3) fenvalerate, (4) etofenprox, and (5) silafluofen. The chromatogram was obtained at the LOD level (0.02 ng mL^−1^).

**Figure 7:**
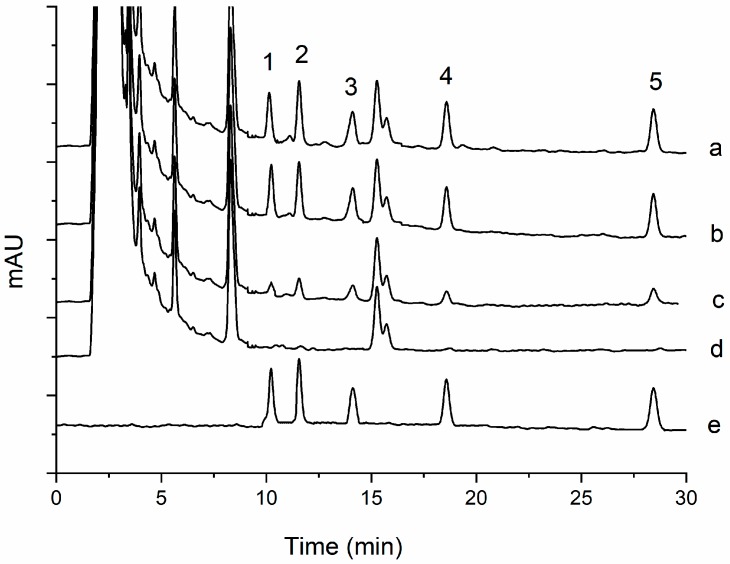
Typical chromatograms of five pyrethroids in oral yimucao liquids under optimum conditions: (1) transfluthrin, (2) fenpropathrin, (3) fenvalerate, (4) etofenprox, and (5) silafluofen. In chromatograms (a, c, d), the spiked levels are 100, 20, and 0 ng mL^−^^1^, and (b, e) show the standard solution in blank matrix and acetonitrile.

**Figure 8:**
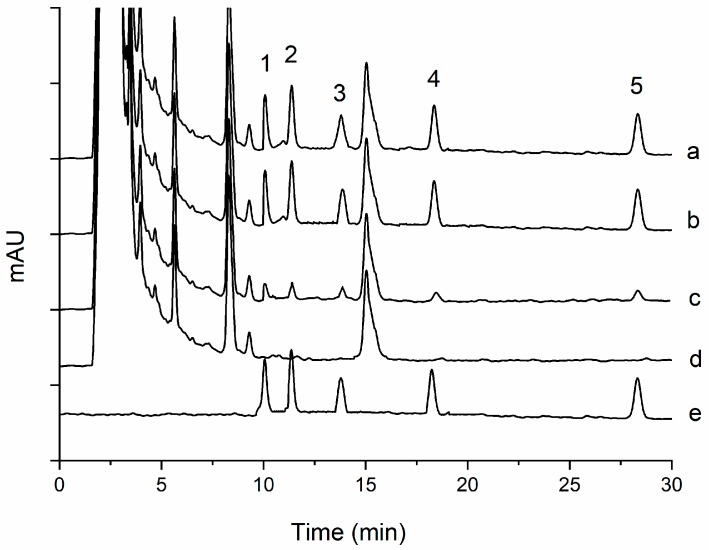
Typical chromatograms of five pyrethroids in oral shengmaiyin liquids under optimum conditions: (1) transfluthrin, (2) fenpropathrin, (3) fenvalerate, (4) etofenprox, and (5) silafluofen. In chromatograms (a, c, d), the spiked levels are 100, 20, and 0 ng mL^−1^, and (b, e) show the standard solution in blank matrix and acetonitrile.

As shown in Figure 5, Figure 6, Figure 7 and Figure 8, all the pyrethroids in the three matrixes could be completely separated in 30 min. The results show that the present method is quite reliable for the determination of the five pyrethroids in the TCM oral liquid samples.

### 2.3. Matrix Effect

Three pesticide-free TCM oral liquids named Qutanling (contains decanoyl acetaldehyde, lauric aldehyde, aspartic acid, salicylic acid, microelement saccharose, sodium benzoate, etc.), Yimucao (contains leonurine, stachydrine, prehispanolone, lavender foliosider, sodium saccharin, etc.), and Shengmaiyin (contains panaxoside, panacen, camphene, sitosterol, schisandrin, saccharose, sodium benzoate, etc.) were tested. The components of a TCM oral liquid matrix are quite complex. This has a significant effect on the signal intensity during the analysis; therefore different matrix-matching calibrations were used. The matrix effect was estimated in the organic solvent and the actual samples by comparing the slopes of calibration curves [18]. Table 3 shows the evaluation of the matrix effect (ME) investigated for five pesticides in three TCM oral liquids. The ME can be considered insignificant when the value obtained is within the range of −20% to 20% [19]. Thus, the results show that no remarkable difference between organic solvent and actual sample were observed. The matrix effect is not significant when using the modified QuEChERS method.

### 2.4. Determination of TCM oral Liquid Samples

Shengmaiyin Oral Liquid, Yimucao Oral Liquid and Qutanling Oral Liquid purchased locally were analyzed using the developed QuEChERS method. The five pyrethroid pesticides were not detected in any of the three TCM oral liquid samples. The chromatograms of the three samples are shown in Figure 5d, Figure 7d, and Figure 8d. Up to now, there have been no limits for pyrethroid residues allowed in TCM oral liquid samples, and the limit for Deltamethrin, which is another kind of pyrethroid, is 20 ng mL^−1^ in the water quality standards for urban water supply (Standards for Urban Construction Industry of the People’s Republic of China: CJ/T 206-2005). The limits of Deltamethrin are higher than the current LOQ values in this work; thus, the proposed method will be a promising tool for the monitoring of pyrethroid residues in the TCM oral liquid samples.

### 2.5. Comparison of Present Method with Other Methods

Table 4 presents a comparison of the current analytical method with the published methods (DLLME, MSPE, and UA-DLLE) for the analysis of pyrethroids in liquid samples [8,20,21]. Although the sample matrix of the TCM oral solution is very complex, the LOQ values are 0.022–0.057 ng mL^−1^, which are slightly better than those of other methods. Besides this, the extraction solvent is more environmentally friendly, and the recovery rate is also comparable with those of other methods. Because the current method has no special equipment requirements, it is promising for the routine analysis of pyrethroids in TCM oral liquid preparations.

## 3. Materials and Methods

### 3.1. Reagents and Materials

**Figure 9:**
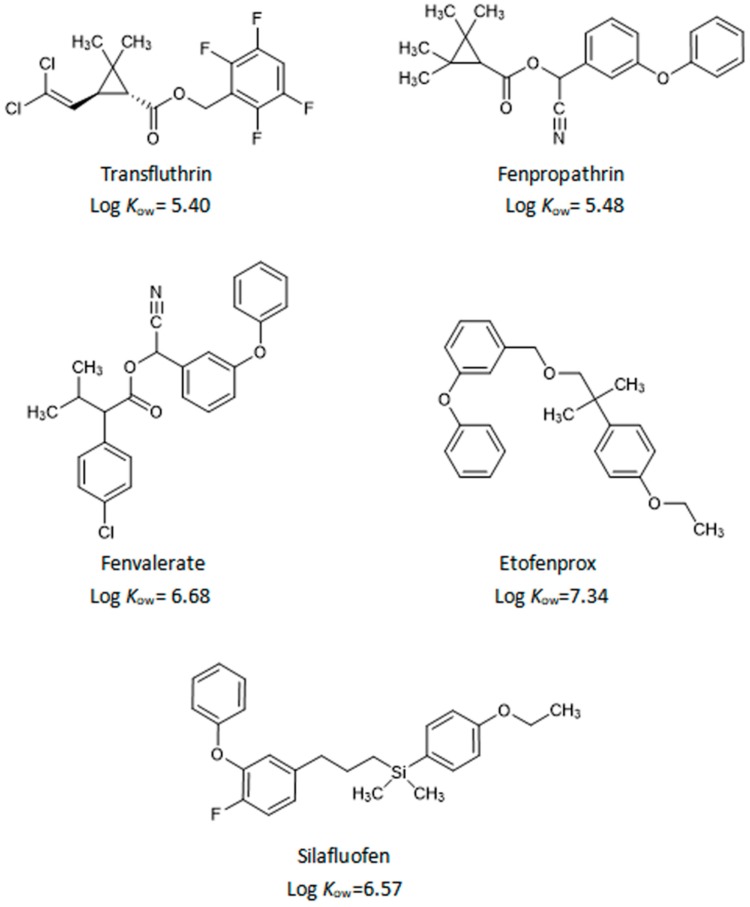
Chemical structures and Log *K*_ow_ values of the five pyrethroids used in the study.

All pyrethroid pesticide standards (transfluthrin, fenpropathrin, fenvalerate, etofenprox, and silafluofen) were purchased from Dr. Ehrenstorfer GmbH (Augsburg, Germany). The chemical structures of the analytes are presented in Figure 9. Sodium chloride (NaCl), anhydrous sodium acetate (CH_3_COONa), anhydrous magnesium sulfate (MgSO_4_), and acetone (CH_3_COCH_3_, HPLC grade) were purchased from Guangzhou Chemical Reagent Factory (Guangzhou, China). Glacial acetic acid (CH_3_COOH, HPLC grade) was obtained from Xiya Chemical Industry Co. (Shandong, Linyi, China). Acetonitrile (CH_3_CN, HPLC grade) was supplied by Mreda Corporation ((Beijing, China). Water was obtained from a Milli-Q water purification system (Millipore, Bedford, MA, USA), and extraction was performed using Agilent SampliQ QuEChERS AOAC Extraction Kits (PN: 5982-5122) [22], Dikma ProElut C18 (PN: 63102, 500 mg/6 mL), and Dikma ProElut NH2 (PN: 63302, 100 mg/1 mL). The PSA (*N*-propylethylenediamine) sorbent was obtained from CNW Technologies (Dusseldorf, Germany). An ultrasonic generator (KQ2200DE, Kunshan Ultrasonic Instruments Co., Ltd., Kunshan, China) with an output power of 100 W and frequency of 40 kHz was used. Qutanling oral liquid (composed of Succus bambusaes and *Houttuynia cordata* Thunb) was provided by Jingan Pharmaceutical Co., Ltd. (ShangHai, China). Yimucao oral liquid (composed of *Leonurus japonicus* Houtt.) was provided by Jinma Pharmaceutical Co., Ltd. (YiYang, China). Shengmaiyin oral liquid (composed of *Codonopsis pilosula* (Franch.) Nannf., *Ophiopogon japonicus* (L. f) Ker-Gawl., and *Schisandra chinensis* (Turcz.) Baill.) was provided by Yunnan baiyao Co., Ltd. (Kunming, China).

### 3.2. Chromatographic Conditions

The LC analysis was performed on a Waters HPLC system (Waters Corporation, Milford, CT, USA) equipped with a 1525 HPLC pump and a 2489 UV/visible detector. The chromatographic separation was carried out on a Diamonsil C18 column (150 × 4.6 mm, 5 μm, Dikma, Beijing, China), which was kept at 30 °C in the column heater (Waters 1500-series column heater). Acetonitrile/water (5:95, *v*/*v*) and acetonitrile/water (95:5, *v*/*v*) were used as mobile phases A and B, respectively, and the total flow rate was 0.6 mL min^−1^. The gradient elution program was as follows: 88% B from 0 to 9 min, 88–100% B from 9 to 30 min, and 100–88% B from 30 to 31 min. The total analysis could be finished in 35 min. The injection amount was 10 μL, and the detection wavelength was 210 nm (the maximum absorption wavelengths of transfluthrin, fenpropathrin, fenvalerate, etofenprox, and silafluofen are 215 nm, 204 nm, 225 nm, 205 nm and 215 nm, respectively).

### 3.3. Optimization of QuEChERS Extraction Method

The effects of extraction parameters, including the extraction solvent (acetonitrile–acetone (70:30, *v*/*v*) solution, acetonitrile containing 1% acetic acid, or acetonitrile), extraction solvent amount (2, 5, or 8 mL), purification agent (PSA and graphitized carbon black, ProElut NH2, ProElut C18, or QuEChERS Extraction Kits), and salt type (CH_3_COONa or NaCl), on the recoveries were investigated by single-factor experiments. The experiments were all carried out in triplicate, and the recovery was calculated using the following equation.

$$R=\frac{{[\mathrm{analyte}]}_{\mathrm{sample}\;\mathrm{with}\;\mathrm{spike}}-{[\mathrm{analyte}]}_{\mathrm{sample}\;\mathrm{without}\;\mathrm{spike}}}{{\left[\mathrm{analyte}\right]}_{\mathrm{added}}}\times 100\%$$

### 3.4. Sample Preparation Procedure

The TCM oral liquid preparations were centrifuged at 8000 rpm for 30 min and the supernatant was filtered through a membrane filter (0.22 μm) before performing the QuEChERS procedure. Five milliliters of 1% acetic acid in acetonitrile were added to 5 mL of a TCM oral liquid in a 15 mL centrifuge tube. After vortexing for 30 s, 2.0 g of MgSO_4_ and 0.5 g of CH_3_COONa were added, shaken for 1 min, and centrifuged at 7000 rpm for 5 min. The obtained supernatant was transferred to QuEChERS Kits (PN: 5982-5122; containing 50.0 mg of PSA, 5.0 mg of C_18_, and 150.0 mg of MgSO_4_) in a 15 mL centrifuge tube. After shaking for 1 min, centrifugation (7000 rpm, 5 min) was performed. The entire supernatant was filtered with a 0.22 μm microporous filter, then dried at 40 °C under a slow stream of nitrogen gas. Finally, it was diluted with 100 μL of acetonitrile, and 10 μL of the resultant solution was injected into the HPLC system.

### 3.5. Method Validation

The selectivity of the method was evaluated by analyzing TCM oral liquid (six independent samples in three cases analyzed separately) and by visual assessment of the baseline at the retention time of analytes [23]. The analytical method was validated by evaluating the limit of detection (LOD), limit of quantitation (LOQ), linearity, matrix effect, precision, and accuracy [5]. The matrix effect was studied by comparing the calibration curves prepared in solvent and in the matrix. Linearity was assessed using curves prepared with pyrethroid concentrations of 0.05, 0.2, 1, 2, 4, 10, 20, 40, 100, and 200 ng mL^−1^ in a blank matrix. The blank matrix was pesticide-free TCM oral solution, which was extracted and purified by the QuEChERS method. The accuracy, in terms of recovery, was evaluated using TCM oral liquid blank samples spiked at 20, 50, and 100 ng mL^−1^ after analyzing three replicates at each concentration. Intra-day and inter-day precisions were evaluated by analyzing three spiked samples (20 ng mL^−1^) five times in a day and on five different days, respectively.

### 3.6. Matrix Effect

The matrix effect greatly affects the accurate detection of target compounds in a complex sample. Many methods have been applied to reducing the matrix effect such as the matrix purification method and the special injection method [24,25,26]. However, some methods are difficult to apply due to the higher requirements of instruments and facilities. The matrix-matched calibration method has been commonly utilized because it is more accurate and simpler [27]. Therefore, it was applied in this research to eliminate the matrix effect of the TCM oral liquid samples. Standard solutions and sample solutions were prepared using a blank matrix that contained no pesticide for the elimination of the matrix effect. The influence of the matrix effect was estimated using the equation [28]:

$$\mathrm{Matrix}\;\mathrm{effect}\;(\%)\;=\frac{X_{1}{-X}_{2}}{X_{2}}\times 100\%$$

where X_1_ is the slope of the curve prepared in the matrix and X_2_ is the slope of the curve prepared in the solvent.

### 3.7. Enrichment Factor

The enrichment factor (EF) is the ratio of the final concentration in the sediment phase (C_fin_) to the initial target component concentration in the TCM sample. The initial concentration was 100 ng mL^−1^. The equation is presented as follows:

$$\mathrm{EF}=\frac{C_{\mathrm{fin}}}{C_{\mathrm{ini}}}$$

## 4. Conclusions

In this study, the QuEChERS method coupled with HPLC was validated for the determination of pyrethroid pesticides in TCM oral liquids. The extraction conditions were optimized, and 5 mL of acetonitrile containing 1% acetic acid was used as the extraction solvent. QuEChERS extraction kits were applied for the purification of the extracts. The chromatographic separation of the pyrethroid pesticides was completed in 30 min. The recoveries of all the pyrethroid pesticides were in the range of 87.20–104.80%. The composition of TCM oral liquids are complex and include Chinese herbal extracts, flavoring agents, bacteriostatic agents, and other components; the QuEChERS method can handle a complex matrix and is very simple and convenient to operate. This simple, low-cost, and highly sensitive analytical method can be a potential tool for the analysis of pyrethroid residues in TCM oral liquid preparations.

## Figures and Tables

**Table 1:** Analytical performance optimized for determination of pyrethroids by QuEChERS method combined with high-performance liquid chromatography and ultraviolet detection (HPLC-UV). LOD: limits of detection; LOQ: limits of quantification.

Samples	Analytes	Linearity Equation	R^2^	Linear Range	LOD	LOQ	Precision (% RSD)		Enrichment Factor
				(ng mL^−1^)	(ng mL^−1^)	(ng mL^−1^)	Intra-Day (*n* = 5)	Inter-Day (*n* = 5)	
Sheng-maiyin oral liquid	Transfluthrin	y = 82.78x + 754.83	0.9991	0.05–200.00	0.011	0.035	3.68	2.61	45.16
	Fenpropathrin	y = 98.49x − 799.59	0.9992	0.05–200.00	0.007	0.022	3.06	2.98	47.67
	Fenvalerate	y = 65.76x + 1722.24	0.9997	0.05–200.00	0.017	0.049	4.62	1.06	47.50
	Etofenprox	y = 79.72x + 762.23	0.9995	0.05–200.00	0.014	0.040	3.60	3.02	48.89
	Silafluofen	y = 87.77x + 2137.18	0.9999	0.05–200.00	0.010	0.032	2.87	2.46	51.07
Qutan-ling oral liquid	Transfluthrin	y = 71.09x − 550.41	0.9997	0.05–200.00	0.016	0.047	3.42	2.73	46.83
	Fenpropathrin	y = 80.01x + 679.88	0.9992	0.05–200.00	0.013	0.038	3.47	1.98	45.84
	Fenvalerate	y = 58.10x + 1250.09	0.9998	0.05–200.00	0.018	0.057	3.17	2.44	47.85
	Etofenprox	y = 74.87x + 951.04	0.9994	0.05–200.00	0.015	0.045	2.79	1.83	46.33
	Silafluofen	y = 79.30x + 1325.92	0.9995	0.05–200.00	0.014	0.042	3.69	2.48	46.73
Yimuc-ao oral liquid	Transfluthrin	y = 81.17x + 499.39	0.9993	0.05–200.00	0.012	0.037	2.68	1.65	50.48
	Fenpropathrin	y = 95.35x − 780.63	0.9999	0.05–200.00	0.008	0.025	3.29	2.66	43.67
	Fenvalerate	y = 71.39x + 140.27	0.9992	0.05–200.00	0.016	0.049	2.62	2.16	45.49
	Etofenprox	y = 79.73x + 1875.92	0.9995	0.05–200.00	0.014	0.043	4.16	2.70	45.77
	Silafluofen	y = 90.96x + 763.42	0.9996	0.05–200.00	0.009	0.029	2.44	1.98	46.98

**Table 2:** Analysis of Traditional Chinese medicine (TCM) samples and spiked recoveries (*n* = 3).

Samples	Spiked Level	Relative Recovery ± RSD (%)				
	(ng mL^−1^)	Transfluthrin	Fenpropathrin	Fenvalerate	Etofenprox	Silafluofen
Shengmaiyin oral liquid	20	93.4 ± 0.9	98.6 ± 1.2	104.8 ± 1.7	99.3 ± 1.9	102.3 ± 1.6
	50	92.7 ± 1.1	95.7 ± 3.6	89.8 ± 2.2	103.9 ± 1.0	98.1 ± 1.4
	100	90.7 ± 1.9	97.4 ± 1.9	93.4 ±1.9	99.1 ± 1.6	104.2 ± 1.3
Qutanling oral liquid	20	89.7 ± 2.3	89.8 ± 2.7	88.8 ± 1.0	89.4 ± 2.3	91.0 ± 1.8
	50	93.9 ± 2.0	89.9 ± 2.4	97.2 ± 1.7	88.1 ± 2.3	99.0 ± 2.4
	100	97.2 ± 1.6	101.0± 2.3	99.4 ± 1.5	101.2 ± 2.1	93.4 ± 0.9
Yimucao oral liquid	20	99.9 ± 0.8	87.5 ± 1.6	88.5 ± 1.1	92.4 ± 2.2	87.2 ± 1.3
	50	98.1 ± 3.2	92.1 ± 2.1	89.0 ± 1.8	92.8 ± 2.2	100.3 ± 2.4
	100	101.7 ± 1.9	83.9 ± 3.9	93.7 ± 1.3	89.1 ± 1.5	98.2 ± 3.4

**Table 3:** Matrix effect of five pyrethroids in different oral liquid samples.

Samples	Matrix Effect (%)				
	Transfluthrin	Fenpropathrin	Fenvalerate	Etofenprox	Silafluofen
Qutanling oral liquid	4.98	−17.21	−15.76	−13.22	−13.22
Yimucao oral liquid	8.78	−2.04	3.06	6.86	7.21
Shengmaiyin oral liquid	8.26	3.78	−6.98	8.24	4.08

**Table 4:** Comparison of the QuEChERS method with other methods for the determination of pyrethroids in liquid samples.

Sample	Extraction Method	Detection Method	Extraction Substance	Sample Amount	LOQ	Recovery (%)	Ref
Water	MSPE	HPLC	MNPs	100.0 mL	0.043–0.087 ng mL^−1^	77.55–92.90	[20]
Human urine	DLLME	HPLC-UV	[BMMIM]TF_2_N	3.00 mL	10.00–40.00 ng mL^−^^1^	87.40–88.70	[21]
Fruit juice	DLLME	HPLC	chloroform	5.0 mL	5.00–10.00 ng mL^−1^	84.00–94.0	[8]
TCM oral liquid	QuEChERS	HPLC-UV	1% Acetic acid/acetonitrile	5.0 mL	0.022–0.057 ng mL^−^^1^	87.20–104.80	This work
